# Vascular Adhesion Protein-1: A Cell Surface Amine Oxidase in Translation

**DOI:** 10.1089/ars.2017.7418

**Published:** 2018-12-11

**Authors:** Marko Salmi, Sirpa Jalkanen

**Affiliations:** ^1^ MediCity, Turku, Finland.; ^2^ Institute of Biomedicine, University of Turku, Turku, Finland.

**Keywords:** leukocyte trafficking, inflammation, cancer, hydrogen peroxide

## Abstract

***Significance:*** Vascular adhesion protein-1 (VAP-1) is an ectoenzyme that oxidates primary amines in a reaction producing also hydrogen peroxide. VAP-1 on the blood vessel endothelium regulates leukocyte extravasation from the blood into tissues under physiological and pathological conditions.

***Recent Advances:*** Inhibition of VAP-1 by neutralizing antibodies and by several novel small-molecule enzyme inhibitors interferes with leukocyte trafficking and alleviates inflammation in many experimental models. Targeting of VAP-1 also shows beneficial effects in several other diseases, such as ischemia/reperfusion, fibrosis, and cancer. Moreover, soluble VAP-1 levels may serve as a new prognostic biomarker in selected diseases.

***Critical Issues:*** Understanding the contribution of the enzyme activity-independent and enzyme activity-dependent functions, which often appear to be mediated by the hydrogen peroxide production, in the VAP-1 biology will be crucial. Similarly, there is a pressing need to understand which of the VAP-1 functions are regulated through the modulation of leukocyte trafficking, and what is the role of VAP-1 synthesized in adipose and smooth muscle cells.

***Future Directions:*** The specificity and selectivity of new VAP-1 inhibitors, and their value in animal models under therapeutic settings need to be addressed. Results from several programs studying the therapeutic potential of VAP-1 inhibition, which now are in clinical trials, will reveal the relevance of this amine oxidase in humans.

## Introduction

Vascular adhesion protein-1 (VAP-1) was discovered already more than 25 years ago, when vascular molecules involved in leukocyte trafficking into inflamed synovium were searched for (108). This line of research stemmed from the finding that lymphocyte adhesion to inflamed synovial vessels was functionally different from those acting in peripheral lymph nodes or mucosa-associated lymphoid tissues (50). We generated antibodies against inflamed human synovial vessels and found that one of the antibodies (1B2) nicely stained the vasculature in synovium. Rather quickly, however, it became evident that 1B2-recognized antigen was not by any means synovium specific. Further expression analyses showed that besides being stored in intracellular vesicles of vascular endothelial cells, it was detected in smooth muscle cells, pericytes, and adipocytes (108). Importantly, on inflammation, 1B2-antigen was translocated from the storage vesicles to the endothelial cell surface, where it mediated leukocyte binding to the vessel wall (113). Based on this property, we named the molecule recognized by 1B2, VAP-1.

## VAP-1: A Unique Cell Surface Expressed Amine Oxidase

**FIG. 1.**
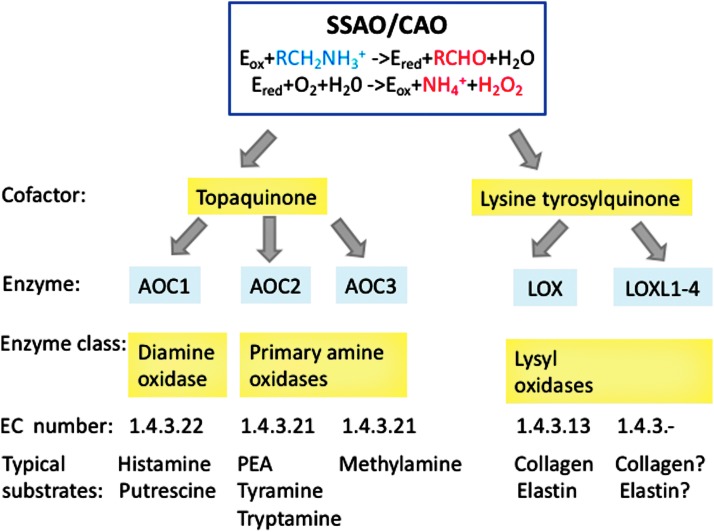
**SSAOs/CAOs oxidate primary amines into aldehydes, ammonium and hydrogen peroxide in a two-step reaction**. SSAOs/CAOs can be classified based on their enzymatic properties (cofactor, substrates) or molecular properties (gene sequences). AOC, amine oxidase copper containing; CAOs, copper-dependent amine oxidases; LOX, lysyl oxidase; LOXL, lysyl oxidase like; PEA, phenyl ethylamine; SSAOs, semicarbazide-sensitive amine oxidases.

In 1990s it was known and well accepted that integrins, immunoglobulin super family members, and selectins were the molecules responsible for leukocyte interaction with vascular endothelium during the multistep extravasation cascade (20, 124). Sequencing of VAP-1 in 1998 therefore brought a big surprise to the field, because both the sequence and the subsequent biochemical work unambiguously showed that in fact VAP-1 is an enzyme catalyzing oxidative deamination of primary amines and producing hydrogen peroxide, ammonium, and aldehydes (123). Similar complementary DNA, cDNA, sequence from human placenta had been independently reported earlier (149). VAP-1 was the first molecularly defined species in the semicarbazide-sensitive amine oxidase (SSAO; EC.1.4.3.6) family, and based on the gene nomenclature, VAP-1 is now officially called as amine oxidase copper containing (AOC). Currently, VAP-1-like SSAOs are renamed as primary amine oxidase (EC.1.4.3.21) to distinguish them better from other SSAOs (19, 129a) (Fig. 1).

Two other topaquinone-containing amine oxidases are diamine oxidase (AOC1) and retina-specific amine oxidase (AOC2) (Fig. 1). AOC1 is a histaminase regulating histamine and putrescine levels and important in maintaining normal pregnancy. AOC1 deficiency is connected to histamine intolerance (32, 88). AOC2 has a relatively large substrate channel allowing it to catalyze oxidation of different monoamines than AOC3. Thus, AOC2 prefers phenyl ethylamine, tyramine, and p-tryptamine as its substrates, whereas methylamine is a preferential substrate for VAP-1. Although AOC2 messenger RNA (mRNA) is found in several organs, its enzymatic activity can be detected only in the eye (52).

Other SSAOs belong to the lysyl oxidase (LOX) family, the members of which have lysine tyrosyl quinone instead of topaquinone at the catalytic site (Fig. 1). LOX activity is important in remodeling of the extracellular matrix. By oxidative deamination of peptidyl lysine residues in collagens and elastin, it triggers their covalent crosslinking. Aberrant expression of LOX has been associated to various diseases. For example, diminished LOX activity is found in certain connective tissue disorders and LOX is increased in liver cirrhosis and Alzheimer's disease (32). Its importance in cancer spread has been recently recognized (22).

The chemistry of the SSAO reaction has been dissected in detail (40, 60). In brief, in the reductive half-reaction of the two-step reaction, a protonated primary amine first interacts with the oxidized form of topaquinone (a post-translational modification of a specific tyrosine residue in VAP-1). The active site base then catalyzes proton abstraction leading to the formation of a product Schiff base and reduced topaquinone. In the subsequent hydrolysis step, a product aldehyde is released. In the second oxidative half-reaction, molecular oxygen reoxidizes topaquinone with concomitant production of ammonium and hydrogen peroxide (Fig. 1).

Overall, SSAOs are relatively well conserved during the evolution and are present even in certain bacterial species/strains such as *Acinetobacter*, *Klebsiella*, *Shigella*, and *Escherichia coli*. Based on the recent analyses of *E. coli* amine oxidase, it is proposed that this hydrogen peroxide-generating enzymatic activity may provide a growth advantage to *E. coli* over other bacteria, which are not able to handle hydrogen peroxide in their living environment (26).

VAP-1 protein is a type 2 transmembrane molecule with a short (in man, only four amino acid long) N-terminal intracellular tail. It is a heterodimer of about 180 kDa and has extensive carbohydrate modifications. A monomer of VAP-1 contains six potential N-linked and three O-linked glycosylation sites and an SSSS sequence as a putative attachment site for additional O-glycans (87).

**FIG. 2.**
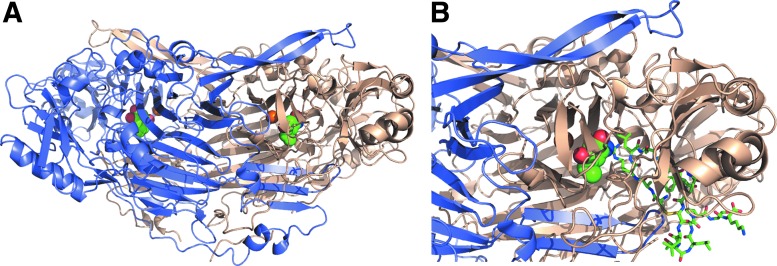
**Crystallographic structure of VAP-1**. **(A)** Two identical monomers are colored *blue* and *wheat*. Copper (Cu) ion is *orange* and TPQ in each chain is presented as *green spheres*. **(B)** Docking of Siglec-9 peptide (*green*) into the active site of VAP-1. This binding mode is presented in Aalto *et al.* (1) and assumes that the peptide binds covalently to TPQ. Courtesy of Dr. Tiina Salminen. Siglec, sialic acid-binding immunoglobulin-type lectins; TPQ, topaquinone; VAP-1, vascular adhesion protein-1.

The crystal structure of VAP-1 has been determined by three groups (29, 48, 102). The extracellular part of human VAP-1 contains three distinct domains (D2–D4) and has an overall heart-shaped structure common to the more primitive SSAOs (Fig. 2). The protein consists of two monomers each with one copper atom. D2- and D3-domains share the same fold consisting of beta-strands and alpha-helices. The large D4-domain is the catalytic domain containing the topaquinone modification and the residues involved in its positioning, the catalytic base, and the copper coordinating histidines. Several intradomain and interdomain cysteines help to stabilize the VAP-1 structure. Large cavities are found both at the dimerization interface and at the active sites. The shape of the active site cavity is determined by several amino acid residues from different domains.

Thus, several D3 residues shape one wall of the active site cavity together with a long β-hairpin arm from D4-domain of the other subunit. Residues from the D4-domain, with some contribution from D2, form the opposite wall of the cavity. Finally, the bottom of the active site cavity is formed by D4-domain residues. The circular shape of the active site cavity critically determines the substrate specificity of VAP-1 by restricting the accessibility of amines to the catalytic site. Moreover, there seems to be a particular “guardian” amino acid at the orifice of the cavity (Leu469 in human VAP-1), the conformation of which may block the entry of potential substrates. The crystal structure also shows that all potential N-glycosylation sites are indeed glycosylated in VAP-1.

The physiologically most relevant soluble substrates of VAP-1 in the body have not yet been identified but at least methylamine and amino acetone can be oxidatively deaminated by VAP-1 (78). These VAP-1 substrates are produced during the intermediary cellular metabolism, and these and many other primary amines can also be ingested in the food or inhaled in the air. The long search for leukocyte ligands of VAP-1 finally resulted in a discovery revealing that sialic acid-binding immunoglobulin-type lectins, Siglec-10, present especially on B cells and monocytes, and Siglec-9, preferentially expressed on monocytes and neutrophils, can bind to VAP-1. Siglec-10 seems to act also as a substrate for VAP-1, but such a function has not been shown for Siglec-9 (1, 59).

## Distribution and Regulation of VAP-1

**FIG. 3.**
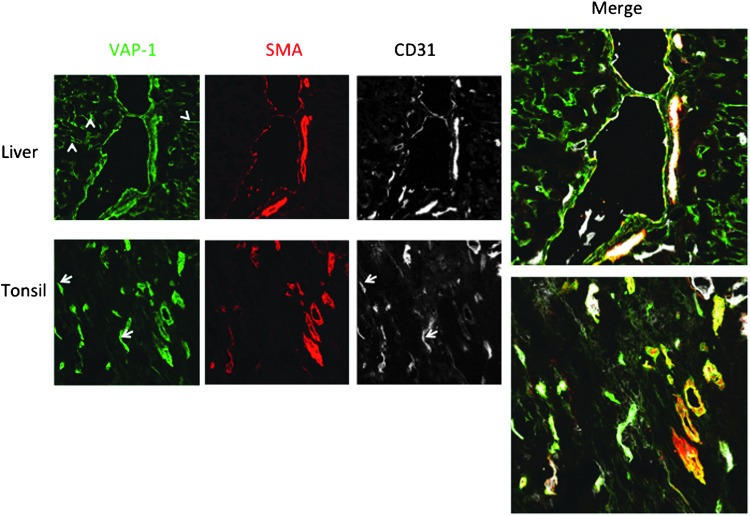
**VAP-1 is expressed in vascular endothelium and smooth muscle in human liver and tonsil.** Triple stainings using anti-VAP-1, anti-smooth muscle actin (SMA), and anti-CD31 (pan-endothelial antibodies) are shown. Note that liver sinusoids (*arrowheads*) stain brightly with anti-VAP-1 but are devoid of smooth muscle present in larger vessels. Similarly, smaller capillaries are VAP-1/CD31 positive but lack SMA in tonsil (*arrows*).

Under normal conditions, VAP-1 is highly expressed in three cell types in humans: vascular endothelial cells, smooth muscle cells, and adipocytes (108, 113). In the vasculature, VAP-1 protein is mainly localized in the cytoplasmic vesicles of endothelial cells throughout the body (113). VAP-1 is present in all three types of endothelial cells. Thus, it is found in continuous (most vessels), fenestrated (*e.g.*, kidney peritubular capillaries), and sinusoidal (liver and bone marrow) endothelial cells, and often more on the venous than capillary or arterial side (Fig. 3) (113). VAP-1 is also strongly expressed in smooth muscle, but not in skeletal or cardiac muscle cells (46). In smooth muscle cells, VAP-1 is enriched in the caveolae of the plasma membrane (46). Also, pericytes lining the outer surface of blood vessels produce VAP-1 (85). In adipocytes, 25% of VAP-1 is found in GLUT4^+^ vesicles (95). During the fetal development, VAP-1 is induced early on in the vasculature and adipocytes (111, 131). VAP-1 is also expressed on dendritic cells in the germinal centers (but not on other leukocyte types), and in chondrocytes (31, 113).

On inflammation, VAP-1 expression in endothelial cells is induced, and the protein translocates from the cytoplasmic vesicles to the plasma membrane (47, 113). The individual inflammatory mediators directly upregulating VAP-1 remain unknown, but in tissue explants, several inflammatory mediators, such as interleukin (IL)-1, tumor necrosis factor (TNF), interferon (IFN)-γ, and lipopolysaccharide (LPS), increase VAP-1 protein expression *ex vivo* (8). In fat cells, VAP-1 is induced during adipocyte differentiation and by TNF *in vitro* (91, 92, 95). So far nothing is known about the regulation of VAP-1 in smooth muscle cells. One possibility is that a splice variant that is a carboxy terminally truncated isoform of VAP-1 lacking several of the amino acids important in the formation of the enzymatic groove of VAP-1 is heterodimerizing with the full-length VAP-1 and thus regulates the expression of the full-length VAP-1 (53).

In general, the expression pattern of VAP-1 seems to be quite similar in human, mouse, rat, and rabbit. However, there are few important differences between the human and mouse, which are the two species used in most VAP-1 studies. Thus, in contrast to humans, the lung vasculature and sinusoidal endothelial cells in the liver express only very low levels of VAP-1 protein in mouse (13).

## Functions of VAP-1

**FIG. 4.**
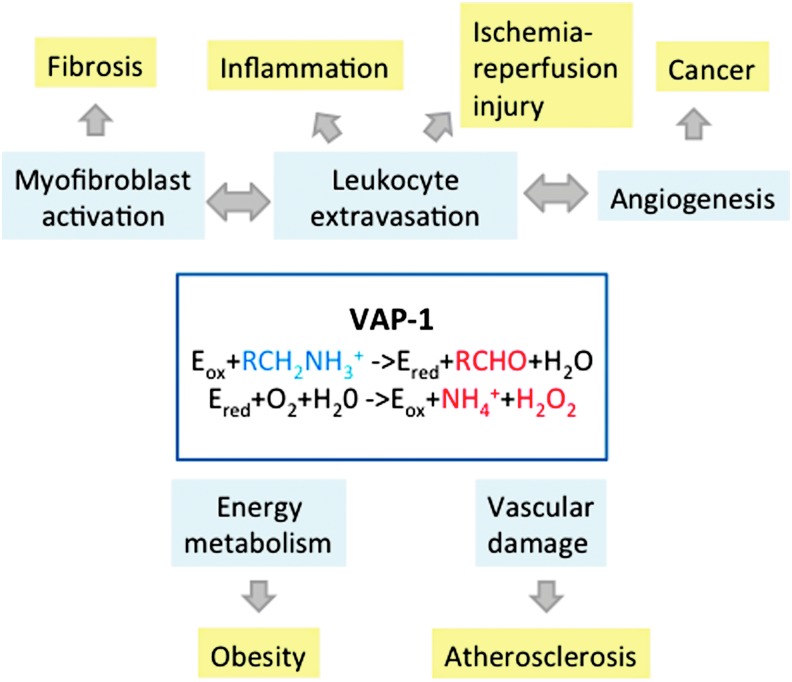
**Functions of VAP-1 in health and disease.** VAP-1 has multiple different physiological functions (*blue boxes*), and it is involved in their aberrations during different disease states (*yellow boxes*). Many of these processes are interdependent, and the role of VAP-1 in leukocyte extravasation is likely to contribute heavily to many of them.

VAP-1 is a truly multifunctional molecule (Fig. 4). The physiological functions of VAP-1 have been dissected most thoroughly in the endothelial cells. After the discovery of the inhibition of lymphocyte binding to high endothelial venules (HEV) by anti-VAP-1 monoclonal antibody (mAb) 1B2, VAP-1 has been shown to mediate leukocyte adhesion to vessels in several *in vitro* binding assays. Thus, human CD4^+^ helper T cells, CD8^+^ cytotoxic T cells, B lymphocytes, monocytes, and granulocytes all bind to HEV and/or flat-walled vessels in different tissues using partially VAP-1-dependent mechanisms (7a, 45a, 89, 107, 109, 113, 114, 116). The dominant role of VAP-1 in lymphocyte binding in liver sinusoidal vessels, which lack most of the traditional adhesion molecules, is intriguing (36).

The contribution of VAP-1 to the leukocyte extravasation has been confirmed in multiple flow-based models. When leukocyte binding to cultured monolayers of VAP-1 expressing endothelial cells isolated from different sources (rabbit heart, human liver) is studied under physiologically relevant laminar shear stress, several anti-VAP-1 antibodies interfere with the multistep adhesion cascade (25, 65a, 117). Notably, also when the enzymatic activity of VAP-1 is blocked using small-molecule SSAO inhibitors, the leukocyte extravasation is compromised under flow conditions (65a, 117). In flow assays, VAP-1 mainly mediates the transmigration step of the extravasation cascade with some effects also on the rolling step and the firm adhesion phase. The flow assays have also recapitulated and expanded the original observations that multiple leukocyte subtypes take benefit of VAP-1 during the exit. For instance, T-reg cells (121), Th17 cells (105), and CD16^+^ monocytes (9) all utilize VAP-1-dependent mechanisms in binding to liver sinusoidal endothelial cells under flow conditions.

The flow assays have also been instrumental in dissecting the role of adhesive and enzymatic functions of VAP-1 in the leukocyte binding. Using human umbilical vein endothelial cells (HUVECs) transfected to express the wild-type VAP-1, Koskinen *et al.* have demonstrated that the anti-VAP-1 antibodies do not interfere with the enzymatic activity of VAP-1, and conversely, the SSAO inhibitors do not affect the expression or antibody epitopes of VAP-1 (63). Both anti-VAP-1 antibodies and SSAO inhibitors inhibit granulocyte rolling and transmigration to the same extent, and their effects are not additive. In addition, when HUVECs are transfected with an enzyme-inactive mutant of VAP-1 (a single amino acid substitution Y471F not affecting the expression or overall structure of VAP-1), leukocyte interactions are significantly reduced when compared with the interactions with HUVECs transfected with the wild-type VAP-1.

These observations have given a strong support to the prevailing model of dualistic VAP-1 functions. Thus, on one hand, VAP-1 appears to contribute to leukocyte binding as an adhesive protein, which can be inhibited by antibodies, in an enzyme activity-independent manner. On the other hand, VAP-1 promotes leukocyte adhesion as an enzyme, the functions of which are dependent on the oxidase activity of the protein (112). In subsequent studies, the catalytic activity of SSAO has been shown to induce the expression of several other adhesion-related proteins in the endothelial cells. Thus, the VAP-1 oxidase activity triggers the synthesis of endothelial adhesion molecules ICAM-1, MadCAM-1, E-selectin, and P-selectin, induces the secretion of chemokine CXCL8, and activates transcription factors such as NF-κB (49, 66, 76).

Many of these responses are dependent on the generation of VAP-1-derived H_2_O_2_, which is a powerful signaling molecule at low concentrations, and are mediated *via* PI3K, MAPK, and NF-κB signaling pathways. Hence, accumulating evidence suggests that the enzymatic activity of VAP-1 triggers autocrine and paracrine signaling cascades, which lead to functional upregulation of many of the classical components of the adhesion cascade.

**FIG. 5.**
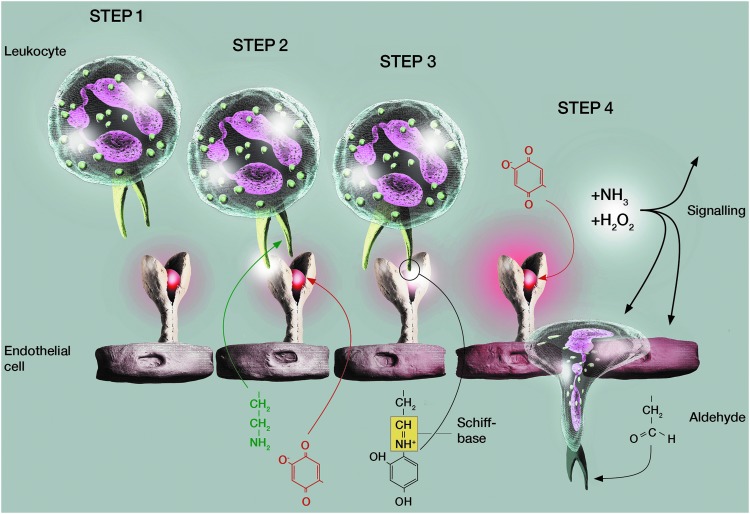
**A working model for VAP-1 function in the leukocyte extravasation cascade.** A blood-borne leukocyte makes sequential contacts with the endothelial cell expressing VAP-1. When the two cell types **(STEP 1)** come in contact with each other **(STEP 2)**, a leukocyte counter-receptor of VAP-1 interacts in an enzyme activity-independent manner with the endothelial VAP-1. Thereafter, the same (or another) leukocyte surface molecule is used as a substrate in the VAP-1-mediated oxidative deamination reaction **(STEP 3)**. This results in the formation of a covalent but transient binding between the two cell types. After the catalytic reaction, the leukocyte surface molecule is modified into an aldehyde, a signaling molecule hydrogen peroxide is formed, and VAP-1 enzyme is converted back to the original state **(STEP 4)**. Note that the order of the proposed **STEPs 2** and **3** is hypothetical.

Siglec-10 and likely other unknown leukocyte ligands of VAP-1 can also serve as substrates for VAP-1 (1, 59). Therefore, we have postulated that VAP-1 may first interact with a leukocyte using an antibody-defined epitope (110, 112). Then, the same (or other) leukocyte surface molecule can subsequently serve as a substrate for VAP-1. This would lead to the formation of a transient Schiff base, which would physically bring together the substrate-expressing cell (the leukocyte) and the enzyme-expressing cell (the endothelium). When the enzyme reaction proceeds, the two cells would spontaneously become separated again with the concomitant formation of H_2_O_2_ and the ensuing signaling effects (Fig. 5). Carbohydrates critically regulate the function of VAP-1, because deglycosylation or mutations of the glycosylation sites on top of the molecule decrease VAP-1-mediated cell adhesion and increase the enzymatic activity of VAP-1 (87, 109).

The *in vivo* relevance of the adhesive role of VAP-1 in the leukocyte traffic has been shown using intravital videomicroscopy. In the early studies, anti-VAP-1 antibodies have been shown to inhibit lymphocyte extravasation in mesenteric venules, to increase granulocyte rolling velocity, and to diminish their transmigration through the cremaster vessels (116, 128). SSAO inhibitors also abolish chemokine-triggered increase in granulocyte rolling and adhesion (120).

The generation of VAP-1 knockout mice (AOC3^−/−^) finally has allowed the formal demonstration of VAP-1 function during the extravasation cascade (126). In VAP-1 knockout mice, the granulocyte rolling velocity is increased and the transmigration efficacy is reduced in the cremaster vessels when compared with wild-type mice. This genetic model also confirms that the effects of the anti-VAP-1 mAbs on the leukocyte extravasation in the wild-type mice are specific, since the antibodies have no effect in the VAP-1 knockout mice. The lymphocytes in VAP-1 knockout mice also display faster rolling behavior in the Peyer's patches (126), and the knockout mice have diminished numbers of lymphocytes in Peyer's patches (62), but in general, the constitutive lymphocyte recirculation through different secondary lymphatic organs is largely intact in the absence of VAP-1.

Collectively, these studies have confirmed that VAP-1 is involved in leukocyte extravasation. The findings have profoundly changed the paradigm of the multistep adhesion cascade by showing that a cell surface expressed ectoenzyme has a major impact on this process, which has earlier been thought to be mediated solely by classical adhesion molecules (selectins, immunoglobulin superfamily members) (110). Later on, many other ectoenzymes have been found to regulate leukocyte extravasation as well.

The physiological functions of VAP-1 on other cell types remain much less thoroughly characterized. On adipocytes, VAP-1 activity is involved in translocation of the glucose transporter GLUT-4 to the cell surface and in insulin-independent glucose uptake (28). It also has insulin-like effects by inhibiting lipolysis and contributes to insulin signaling (27, 96). However, this pathway seems to play only a minor role under physiological conditions, as VAP-1 knockout mice are normoglycemic, leaving the normal metabolic activities of VAP-1 to be dissected in detail.

The smooth muscle VAP-1 does not mediate lymphocyte binding under *in vitro* conditions (46), making it unlikely that pericyte VAP-1 would be involved in the lymphocyte transmigration through the pericyte layer of the vessel wall *in vivo*. Although transgenic overexpression of VAP-1 in smooth muscle cells has been reported to manifest with abnormal aortic structure and hypotension (35), VAP-1 knockout mice are normotensive and show completely normal mechanical properties of the aorta (93). Therefore, the physiological functions of VAP-1 in smooth muscle cells currently remain unknown.

## VAP-1 as a Therapeutic Target in Preclinical Models

The significant, but not irreplaceable, role of VAP-1 in leukocyte extravasation has rendered it an attractive target for therapeutic modulation of inappropriate inflammatory reactions. Aberrant leukocyte migration is a key pathogenic event in a multitude of diseases ranging from classical autoimmune disorders and infections to ischemia/reperfusion, cancer, and fibrosis. The adhesive function of VAP-1 can be inhibited either by monoclonal antibodies or by small-molecule SSAO inhibitors (Table 1). The potency of the inhibitors varies and their selectivity has been tested using various amines. For example, semicarbazide, widely used as an inhibitor in animal models, also inhibits LOXs and is therefore not optimal (90).

**Table 1.** Selected Small-Molecule Vascular Adhesion Protein-1 Inhibitors

*Inhibitor*	*Name*	*Group*	*IC_50_*	*Company*	*References*
SC	Semicarbazide				
HA	Hydroxylamine				
LJP1207	N′-(2-phenyl-allyl)-hydrazine	Hydrazine	17 n*M*	La Jolla	(118)
LJP1586	Z-3-fluoro-2-(4-methoxy-benzyl) allylamine	Amine	43 n*M*	La Jolla	(103)
BTT2027	Trans-2-(1methyl-hydrazino)-1-indanol	Indanol	600 n*M*	Biotie	(63)
BTT2052	(*1S,2S*)-2-(1-methyl-hydrazino)-indanol (dibasic)	Indanol	400 n*M*	Biotie	(86)
SZE5302	Cis-isomer of BTT2052				
PXS-4681A	(Z)-4-(2-(aminomethyl)-3-fluoroallyloxy)benzene-sulfonamide		3 n*M*	Pharmaxis	(34)
PXS-4159A		Amine	10 n*M*	Pharmaxis	(33)
PXS-4728A	4-(E)-2-(aminomethyl)-3-fluoroprop-2-enoxy)-N-tert-butylbenzamide	Amine	5 n*M*	Pharmaxis	(120)
U-V002		Thiazole	7 n*M*	R-Tech	(98)
SzV-1287	3-(4.5-Diphenyl-1,3-oxazol-2-yl) propanal oxime	Oxime	3.5 μm	Semmelweis Egyetem	(41)

IC_50_, half of the maximal inhibitory effect on human VAP-1.

VAP-1, vascular adhesion protein-1.

The selectivity data concerning the listed inhibitors are only available to a limited extent [reviewed in Dunkel *et al.* (24)]. Both therapeutic avenues, antibody and inhibitor-based ones, have been tested in a multitude of different disease models (Table 2). However, very different experimental designs (including the choice of controls and readouts) and reporting formats with relatively small numbers of animals have been used in VAP-1 studies, which, in most cases, precludes rigorous and meaningful statistical analyses of the variation of the results. Therefore, the effects listed in Table 2 are only meant to give a rough estimate of the translational potential of VAP-1 manipulation, and the reader is referred to the original work for the details of the experimental settings.

**Table 2.** Preclinical Models of Vascular Adhesion Protein-1 Targeting

*Model*	*Species*	*Treatment*^a,b^	*Major effects*^c^	*References*
Autoimmune and other inflammations				
Peritonitis				
PP+IL-1	Rabbit	mAb TK8-14	Leukocyte infiltration reduced by 70%	(128)
PP+IL-1	Mouse	mAb 7-106	Leukocyte infiltration reduced by 40%	(94)
TNF	Mouse	KO	Granulocyte infiltration reduced by 40%	(126)
TNF	Mouse	SSAO KO	Neutrophil infiltration reduced by 50%	(101)
Thioglycollate	Mouse	SSAO KO	No differences in leukocyte infiltration	(101)
Skin inflammation				
Carrageenan	Rat	LJP1207	Paw edema reduced by 70%	(118)
Carrageenan	Rat	BTT2027	Leukocyte infiltration reduced by 50%	(63)
Carrageenan	Mouse	LJP1586	Granulocyte infiltration reduced by 50%	(103)
Carrageenan	Mouse	PXS-4681A	Neutrophil infiltration reduced by 60%	(34)
CCL21	Mouse	mAb 7-106	Monocyte infiltration reduced by 60%	(94)
Lung inflammation				
LPS	Rat	LJP1586	Granulocytes in BAL reduced by 40-60%	(103)
LPS	Mouse	PXS4159A	BAL leukocytes reduced by 60%	(33)
LPS	Mouse	PXS-4681A	Lung neutrophils remain at baseline	(34)
LPS	Mouse	**PXS-4728A**	BAL neutrophils reduced by 25%	(120)
LPS	Mouse	TG mice	BAL neutrophils increased by 25%	(145)
Gut inflammation				
Oxazolone-induced colitis	Mouse	LJP1207	Inflammation and ulceration reduced by 90%, mortality reduced by 50%	(118)
OVA vaccination	Mouse	KO	T cell proliferation reduced by 50%	(62)
Liver inflammation				
ConA	Mouse	mAb 7-88 + 7-106	ALT increase inhibited by 70%	(12)
ConA	Mouse	KO	Hepatic CD4^+^ decreased by 50%	(68)
Eye				
LPS uveitis	Rat	U-V002	Leukocyte infiltration to anterior chamber reduced by 60%	(98)
Type 1 diabetes	Rat	U-V002	Leukocyte transmigration to retina reduced by 40%	(99)
Arthritis				
Adjuvant induced	Rat	BTT2052	Arthritis score decreased by 50%	(86)
CAIA	Mouse	BTT2052	Histological damage reduced by 75%	(86)
	Mouse	KO	Histological damage reduced by 50%	
Serum transfer	Mouse	SzV-1287	Clinical arthritis score reduced by 40%	(41)
	Mouse	LJP-1207	Clinical arthritis scores reduced by 50%	
Adjuvant induced	Mouse	SzV-1287	Histologic arthritis score reduced by 40%	(41)
		LJP-1207	No effect on histological damage	
Neuroinflammation				
MOG	Mouse	LJP1207	Clinical score reduced by 25%	(133)
PLP	Mouse	**LJP1207**	Clinical score reduced by 40%	(104)
LPS	Rat	PXS-4681A	Leukocyte extravasation diminished by 70%	(11)
Autoimmune diabetes				
NOD	Mouse	mAb7-88	Diabetes incidence reduced by 40%	(94)
Allograft rejection				
Liver	Rat	mAb174-5	Lymphoid infiltration reduced by 50%	(81)
Bronchial asthma				
OVA induced	Mouse	KO	No clear effect on pulmonary leukocytes	(23)
Infection				
*Yersinia enterocolitica*	Mouse	KO	Trend of decreased lethality	(126)
*Staphylococcus aureus*	Mouse	KO	Transient small increase in bacterial multiplication	(62)
	Mouse	mAb+BTT2052	No effects on bacterial growth	
Coxsackie B4	Mouse	KO	Slightly increased pancreatic inflammation	(62)
	Mouse	mAb+BTT2052	No effects on pancreatic inflammation	
LPS endotoxemia	Mouse	LJP1207	Survival increased from 40% to 90%	(118)
*Klebsiella pneumoniae*	Mouse	PXS4728A	Airway neutrophils reduced by >50%	(120)
Polymicrobial sepsis (CLP)	Mouse	PXS4728A	Normal peritoneal, but >80% reduced BAL neutrophils	(120)
Rhinovirus + asthma	Mouse	PXS4728A	BAL neutrophils reduced by 50%	(120)
IRI				
Intestinal IRI	Mouse	KO	Intestinal and lung damage decreased by 30%	(58)
Mouse	SZE5302	Intestinal damage reduced by 75%, lung by 25%		
Mouse	mAb 7-106	No effects		
Hemorrhagic	Mouse	LJP1586	Neurologic score improved by 75%	(79)
stroke	Mouse	siRNA	Neurological score improved by 20%	
Cerebral artery	Rat	**LJP1586**	Infarct volume reduced by 30–50%	(134)
occlusion	Rat	**LJP1207**	Neurologic outcome improved by 75%	(140)
Subarachnoidal hemorrhage	Rat	**LJP1586**	Neurologic outcome improved by 25%	(138)
Myocardial infarction	Rat	LJP1207	Infarct size reduction by 40%	(142)
Renal IRI	Rat	RTU-1096	Tubular injury score reduction by 40%	(127)
Fibrosis				
CCL4 induced	Mouse	KO	Liver fibrosis reduced by 50%	(136)
Mouse	mAb BTT1029	Liver fibrosis reduced by 25%		
Methionine-choline-free diet	Mouse	KO	Liver fibrosis inhibited by 75%	(136)
	Mouse	mAb **BTT1029**	Collagen synthesis inhibited by 80%	
Mouse	SSAO-KO	Hepatic CD4^+^ and NKT infiltration reduced by 50%		
Western diet	Mouse	KO	Steatohepatosis inhibited by 50%	(136)
Ureteral obstruction	Mouse	PXS4728A	Renal fibrosis reduced by 40%	(137)
Platinum induced	Mouse	**PXS4728A**	Renal fibrosis reduced by 50%	(55)
Cigarette smoke	Mouse	**PXS4728A**	Complete reversal of lung function defects	(51)
Bleomycin induced	Mouse	KO	Lung fibrosis score reduced by 40%	(85)
Mouse	SSAO-KO	Lung fibrosis score reduced by 50%		
Mouse	**LJP1586**	Lung fibrosis score reduced by 40%		
Cancer				
Melanoma	Mouse	KO	Tumor size reduced by 25%	(83)
Mouse	KO/hTG	Tumor size reduced by 25%		
Melanoma	Mouse	mAb7-106 + 7-88	No effect on tumor size	(84)
Mouse	SZE5302	Tumor size reduced by 30%		
Melanoma	Mouse	PRX.A	Metastatic nodules reduced by 25–50%	(30)
Liver cancer	Mouse	LJP1207	Tumor growth reduced by 50%	(72)
Angiogenesis				
Laser injury	Rat	U-V002	Choroidal neoangiogenesis reduced by 40%	(100)
IL-1 induced	Mouse	U-V002	Corneal neovascularization reduced by 30%	(97)
VEGF induced	Mouse	U-V002	No effect on corneal neoangiogenesis	(97)
Streptozotocin	Mouse	Amino imidazole	Macular edema reduced by 40%	(43)
Melanoma	Mouse	KO	Tumor neoangiogenesis inhibited by 40%	(83)
	Mouse	SZE5302	Tumor neoangiogenesis reduced by 60%	(84)
Metabolism				
Type 1 diabetes	Rat	BENZ	Hyperglycemia reduced by 50%	(82)
Type 2 diabetes	Rat	BENZ	Hyperglycemia reduced by 50%	(4)
Normal	Mouse	TG	Diabetes-like vascular complications	(125)
Type 2 diabetes	Mouse	MDL72974A	Atherosclerotic lesions reduced by 50%	(146)
Type 2 diabetes	Mouse	FPFA	Weight gain reduced by 50%, hyperglycemia reduced by 40%	(147)

^a^ BENZ, benzylamine; KO, VAP-1 knockout mice; mAb, monoclonal antibody; SSAO-KO, enzymatically inactive mouseVAP-1 in knockin mice; TG, transgenic mice overexpressing human VAP-1 on endothelium; KO/hTG VAP-1 knockout mice expressing human VAP-1 on endothelium; FPFA, (E)-2-(4-fluorophenethyl)-3-fluoroallylamine, for the other small-molecule SSAO inhibitors, see Table 1.

^b^ Therapeutic (*i.e.*, mAb/SSAO inhibitor first administered after the onset of disease process in a clinically relevant manner) regimens in bold.

^c^ The best effects approximated from the original graphs.

ALT, alanine aminotransferase; BAL, bronchoalveolar lavage; CAIA, anticollagen antibody-induced arthritis; CLP, cecal ligation puncture; ConA, concanavalin A; IL, interleukin; IRI, ischemia/reperfusion injury; LPS, lipopolysaccharide; MOG, myelin oligodendrocyte glycoprotein; NOD, nonobese diabetic; OVA, ovalbumin; PLP, proteolipid protein; PP, proteose peptone; siRNA, small interfering RNA; SSAO, semicarbazide-sensitive amine oxidase; TNF, tumor necrosis factor.

### Acute inflammation

The first *in vivo* evidence for the anti-inflammatory potential of VAP-1 inhibition was obtained when anti-VAP-1 antibodies were found to decrease leukocyte infiltration by about 70% in an acute model of peritonitis in rabbits (128). These findings have then been reproduced in anti-VAP-1 mAb-treated mice and in VAP-1 knockout mice (94, 126). Moreover, mice harboring an enzymatically inactive VAP-1 protein also show attenuated acute peritonitis on TNF-α instillation (101). This serves as a genetic proof for the notion that the enzyme activity-dependent mode of VAP-1 function is functionally operative *in vivo*. The contribution of VAP-1 to peritonitis may be stimulus dependent, since no effects are seen in thioglycollate-triggered peritonitis models in these mice (101).

In several acute skin inflammation models, anti-VAP-1 antibodies and SSAO inhibitors reduce paw edema, prostaglandin E2 production, and granulocyte and monocyte infiltration (63, 94, 118). Acute LPS-induced lung injury also partially relies on a VAP-1-dependent component inasmuch SSAO inhibitors reduce leukocyte accumulation in bronchoalveolar lavage fluid and TNF-α production (34, 103, 120). This is in line with the aggravated LPS-mediated lung injury in transgenic mice overexpressing human VAP-1 in the endothelium (145). In a concanavalin A (ConA)-induced liver injury model, anti-VAP-1 antibodies inhibit effectively the rolling of Th2 cells in the liver sinusoids, but have no effect on the rolling of Th1 cells at this location (12). Up to 90% of acute granulocyte rolling—but not adhesion—in the inflamed liver is also blocked by anti-VAP-1 antibodies. In the ConA-induced liver injury model, anti-VAP-1 antibodies attenuate disease severity better than α4-integrin blockade (68). Liver damage and infiltration by CD4^+^ cells are also reduced in VAP-1 knockout mice. In models of eye inflammation, an SSAO inhibitor suppresses leukocyte recruitment into the retina and vitreous and anterior chamber in LPS-induced uveitis (98). Similarly, SSAO inhibitors inhibit leukocyte transmigration, but not adhesion, in retinal vessels in streptozotocin-induced model of type 1 diabetes (99). Leukocyte infiltration to the retina is also partially VAP-1 dependent. Thus, blocking of VAP-1 function by neutralizing antibodies or by inhibiting its oxidase activity alleviates acute inflammatory reactions in multiple organ systems.

### Chronic inflammation

VAP-1 modulation has beneficial therapeutic effects also in models of long-term inflammation. We have reported the reduction of the clinical disease score, synovial leukocyte infiltration, and cartilage damage after VAP-1 inhibitor treatment and VAP-1 knockout mice in adjuvant-induced arthritis (duration 28 days) and anti-collagen antibody-induced arthritis (duration 6 days) (86). In a 21-day-long serum transfer and adjuvant-induced models of arthritis, two other SSAO inhibitors diminish hyperalgesia, edema formation, and clinical arthritis scores (41). These inhibitors also reduce the production of chemokine CXCL1 in the joints, and LJP1207 inhibitor decreases the production of cartilage matrix *in vitro* as well. Notably, the other SSAO inhibitor used, ScV-1287, inhibits both SSAO and nociceptive ion channels (106).

VAP-1 inhibition also alleviates chronic inflammation in other organ systems. In a 1-week model of oxazolone-induced colitis, the inflammatory cell infiltrate, production of interleukin (IL)-4, IL-5, and IL-13, ulceration, and mortality are reduced on an SSAO inhibitor treatment (118). VAP-1 knockout mice also show attenuated T and B cell responses to oral immunizations (62). In a multiple scelerosis disease model (relapsing/remitting form of experimental allergic encephalomyelitis, EAE), SSAO inhibitors reduce the clinical disease score and the incidence of the disease when first started at the peak of the disease attack (104). Most notably, when started during the first remission, the VAP-1 inhibitor is still effective in diminishing the severity of relapses.

In a spontaneous mouse model of type 1 diabetes (nonobese diabetic [NOD] mice), a half-year-long administration of anti-VAP-1 antibodies postpones the development of diabetic hyperglycemia (94). In an asthma model, the early T cell recruitment to bronchial lymph nodes is strongly reduced in VAP-1 knockout mice, but this translates only to minor changes in effector leukocyte recruitment to the lungs (23). Also, in a rat liver allograft rejection model, inhibition of VAP-1 with antibodies reduces the accumulation of activated lymphocytes and histological tissue damage (81). Collectively, these data imply that chronic VAP-1 inhibition has therapeutic potential even when started after the disease onset, and that it does not lead to any apparent side effects.

### Infections

The blockade of VAP-1 function with the concomitant inhibition of leukocyte patrolling apparently does not increase the risk of opportunistic infections. Thus, the VAP-1 knockout mice do not show any evidence of increased frequency of spontaneous infections (126). Bacterial proliferation is slightly and transiently enhanced in VAP-1 knockout mice exposed to intradermal inoculation of *Staphylococcus aureus*, but the bacterial control is later similar to the control mice (62). Similarly, pancreatic inflammation on exposure to Coxsackie B virus is mildly enhanced in VAP-1 knockout mice. However, the acute blocking of VAP-1 in wild-type mice with antibodies and SSAO inhibitors during these two infections does not alter the microbial multiplication or inflammatory responses (62). Similarly, a 6-month treatment with anti-VAP-1 antibodies does not lead to any apparent infectious complications (94). VAP-1 knockout mice also cope with an intragastric *Yersinia enterocolitica* inoculation at least as effectively as the wild-type controls (126). In an intranasal *Klebsiella pneumoniae* infection model, SSAO inhibition reduces leukocyte migration to airways and increases bacterial load, but it does not affect the survival rates (120). When a rhinovirus infection is superimposed on an asthma mode, the same researchers have found a diminished neutrophil infiltration and airway hypersensitivity after an SSAO inhibition (120).

On the contrary, therapeutic administration of SSAO inhibitors after an LPS-induced shock leads to improved survival rates (118). Also, in another sepsis-like model (cecal ligation puncture leading to systemic bacteremia), SSAO inhibitors do not decrease local peritoneal inflammation, but do reduce distant lung injury, and show a trend of improved survival rates (120). Thus, lifelong deficiency of AOC3 appears to slightly impair inflammatory responses against mild microbial infections. Short- or long-term therapeutic interference with VAP-1 function has so far shown no evidence of increased susceptibility to microbial infections. In contrast, in severe systemic infections, VAP-1 inhibition may actually protect against triggering of overactive systemic inflammatory responses.

### Ischemia/reperfusion injury

Re-establishment of circulation after myocardial infarction or stroke aggravates the tissue injury due to an inappropriate leukocytic infiltration into the hypoxic area. After transient clamping of the mesenteric artery, both the local ischemic tissue damage in the gut and the distal tissue damage in the lungs are less severe in VAP-1 knockout mice than in wild-type mice (58). The SSAO activity appears to be important in this response, since an SSAO inhibitor, but not anti-VAP-1 antibodies, provides similar protection in intestinal ischemia/reperfusion injury in wild-type mice. The beneficial role of VAP-1 blockade by SSAO inhibitors is also seen in postischemic damage in the brain. In a hemorrhagic stroke model, an SSAO inhibitor treatment improves neurological performance and reduces brain edema, leukocyte infiltration, and cytokine induction (79). The protective effects of the oxidase inhibitor were reversed by administering recombinant VAP-1 as a competing substrate. Modest therapeutic effects in the same model were also observed using VAP-1 small interfering RNA (siRNA) (79).

In a forebrain ischemia/reperfusion model, an SSAO inhibitor treatment improves neurological outcome and reduces leukocyte accumulation (140). In a similar middle cerebral artery occlusion model, a postponed treatment with another SSAO inhibitor reduced infarct volumes and improved neurological functions (134). Intravital analyses of granulocyte trafficking in pial venules show that an SSAO inhibitor blocks, without affecting adhesion, the neutrophil transmigration to the levels observed in neutropenic rats. VAP-1 blockade with an SSAO inhibitor also reduces the neurological consequences of subarachnoid hemorrhage by attenuating leukocyte trafficking to the site of insult and pial arteriolar reactivity to dilatory signals (138, 139). In a myocardial ischemia/reperfusion model, an SSAO inhibitor reduces infarct volume and diminishes leukocyte trafficking to the area of injury (142). In a kidney-ischemia/reperfusion model, SSAO inhibition led to a decreased neutrophil infiltration and tubular damage (127). The beneficial effects of VAP-1 inhibition in ischemia/reperfusion injury likely depend on reduced extravasation of granulocytes, although the direct effects of diminished VAP-1-dependent generation of potentially cytotoxic reactive oxygen species cannot be excluded. In many ischemia/reperfusion models, therapeutic administration of SSAO inhibitors during the reperfusion phase, which is clinically relevant, has shown promising short-term protection.

### Fibrosis

Abnormal leukocyte accumulation is one of the driving forces in fibrotic diseases. In three different liver fibrosis models, including a 9-month western diet model, steatohepatitis and fibrosis are significantly reduced in VAP-1 knockout mice when compared to controls (136). Fibrogenesis is at least partially oxidase activity dependent, inasmuch the mice harboring an enzymatically inactive mutant of VAP-1 are also protected from the disease. The beneficial antifibrotic effects are reproduced by treating the wild-type mice with anti-VAP-1 antibodies. Mechanistic studies show that leukocyte infiltration to the liver is strongly reduced with the most prominent changes in CD4^+^ and NKT cells in the absence of VAP-1 function. Moreover, VAP-1 was strongly induced in the collagen-generating myofibroblasts. Recombinant VAP-1 enhances myofibroblast spreading, serves as a potent chemoattractive signal for myofibroblasts, and induces the transcription of profibrotic factors in an oxidase activity-dependent manner (136).

VAP-1 blockade is also effective in treating renal and lung fibrosis. In a urethral obstruction model, SSAO inhibition diminishes renal fibrosis, leukocyte infiltration, and extracellular matrix protein synthesis (137). In a platinum-induced acute kidney injury model, an SSAO inhibitor treatment in a therapeutic setting also alleviates kidney damage, improves kidney function, reduces oxidative stress, and diminishes the synthesis of profibrotic factors, including TGF-β (55).

In the lungs, SSAO inhibition decreases inflammatory leukocyte infiltration into airways and synthesis of proinflammatory cytokines in a cigarette smoke-induced chronic obstructive pulmonary disease (COPD) (51). Notably, in a COPD model, a postponed SSAO treatment successfully reduces leukocyte infiltration and fibrosis, and improves lung compliance back to the control levels, although it does not affect emphysema-like alveolar enlargement (51). In addition, in bleomycin-induced lung damage, the fibrotic response is less severe in the VAP-1 knockout mice, in mice expressing the enzymatically inactive version of VAP-1, and in wild-type mice treated prophylactically or therapeutically with an SSAO inhibitor (85). Thus, VAP-1 function seems to contribute to fibrogenesis both by regulating the inflammatory response in the damaged organ and by directly modulating the myofibroblast responses to the insulting stimuli.

### Cancer

The ability to avoid immune attack is one of the hallmarks of cancer. Proinflammatory antitumor responses are beneficial in keeping tumor progression under control. However, cancer cells utilize multiple tumor evasion mechanisms, which tame the antitumor immune responses and in fact subvert the functions of the inflammatory cells to tumor-promoting direction (38). Inspired by the findings that tumor infiltrating leukocytes use VAP-1 to bind to tumor vessels *ex vivo* (45, 144), the role of VAP-1 in cancer has been analyzed *in vivo*. In a melanoma model, the tumor progression is delayed in VAP-1 knockout mice and in mice lacking the VAP-1 oxidase function in endothelial cells (83). Leukocyte infiltrates in the tumors show selective reduction of myeloid-derived suppressor cells (MDSC) in the absence of VAP-1, and when the immature myeloid cells capable of suppressing T cells are depleted in mice, the tumor growth is no more retarded in VAP-1 knockout mice (83). Anti-VAP-1 antibodies reduce leukocyte–tumor vessel interactions *in vivo* and diminish the accumulation of cytotoxic cells in the tumor, but do not affect the growth of melanoma (84).

An SSAO inhibitor, in contrast, attenuates tumor progression in melanoma and lymphoma models, and this is associated with diminished infiltration of MDSC into the tumors (84). Similar findings have been reported in a liver carcinoma model using another SSAO inhibitor (72). In addition, an early inhibition of VAP-1 by an SSAO inhibitor during metastatic seeding diminishes the formation of pulmonary metastases on intravenous tumor challenge with melanoma and mammary carcinoma cells (30). This inhibitor treatment leads to reduced infiltration of CD11b^+^ myeloid cells at the initial sites of metastatic deposits. In humans amplification of VAP-1 gene has been found in gastric cancer (132), whereas a decrease of VAP-1 protein in aggressive prostate cancer has been reported (21). Thus, interference of the oxidase activity of VAP-1 in developing tumors may have beneficial growth-inhibiting effects apparently through modulation of the composition of intratumoral leukocyte infiltrate.

### Angiogenesis

VAP-1 inhibition reduces pathological growth of new vessels. While the physiological vasculogenesis and angiogenesis are intact in VAP-1 knockout mice (126), SSAO inhibitors reduce aberrant angiogenesis in several eye disease models, including a laser-induced choroidal damage and a cytokine-induced corneal micropocket assay (97, 100). In mechanistic analyses, the diminished recruitment of myeloid cells (monocytes/macrophages) to the affected vasculature has been shown to be responsible for the therapeutic effects. VAP-1 may exert stimulus-specific effects on the neoangiogenesis; inasmuch IL-1β-induced corneal neoangiogenesis is inhibited by an SSAO inhibitor, whereas VEGF-A-induced is not (97).

These findings may be explained by the fact that the IL-1β-induced infiltration of VEGF-producing monocytes/macrophages, the need for which is circumvented by direct VEGF administration, is strongly VAP-1 dependent. SSAO inhibitors also diminish the leakage of retinal vessels (43). VAP-1-dependent neoangiogenesis may also play a role in cancer. Diminished angiogenic response in growing melanoma tumors is observed in the VAP-1 knockout mice and on SSAO inhibitor treatment (83, 84). Whether the proangiogenic functions of VAP-1 solely depend on its ability to assist the recruitment of VEGF-producing myeloid cells in different settings, or whether endothelial SSAO activity also has direct effects on the neoangiogenic endothelial cells remains to be determined.

### Glucose metabolism

The function of VAP-1 in regulating glucose uptake has been a long-standing interest in translational models. In streptozotocin-induced diabetes, treatment with a VAP-1 substrate benzylamine (in combination with low-dose vanadate) improves glucose tolerance, upregulates GLUT-4 expression on fat cells, and reduces hyperglycemia (82). In a spontaneous type 2 diabetes model in Goto-Kakizaki rats, both acute and chronic administrations of benzylamine (together with vanadate) stimulate glucose utilization in fat and muscle cells, upregulate GLUT-4 expression, and reverse insulin resistance in the muscle (4). This treatment also stimulates insulin secretion. Provision of an SSAO substrate (methylamine, without vanadate) to human and wild-type mice hepatocytes, but not to hepatocytes of VAP-1 knockout mice, increases glucose uptake and GLUT-4 expression in an oxidase activity-dependent manner in *ex vivo* assays (54).

In transgenic mice overexpressing VAP-1 on the endothelial cells, an initial improvement of glucose tolerance is overridden by vascular complications (glomerulosclerosis, atherosclerosis, and hypertension) typical to diabetic patients at later time points (125). When KKAy-diabetic mice fed with cholesterol-rich diet are treated with SSAO inhibitors, a decrease in weight and in atherosclerotic lesions is observed (146, 147). However, VAP-1 knockout mice, although slightly fatter with concomitant diminished leukocyte infiltration in adipose tissue, are normoglycemic, and their glucose tolerance is normal (18). Therefore, elucidation of the *in vivo* metabolic effects after selective and isolated VAP-1 targeting needs to be addressed in future studies.

## Clinical Implications

### Soluble VAP-1 as a biomarker

VAP-1 is found as a soluble form in the blood (64a). Soluble VAP-1 (sVAP-1) is a cleavage product of the membrane-bound VAP-1 and liver sinusoidal endothelial cells are one source of it in man (65). In healthy individuals, the concentrations of the soluble form of VAP-1 stay rather stable, but in several diseases either increased or decreased levels have been reported (Table 3). Inflammation *per se*—as evidenced by the lack of correlation between C-reactive protein, CRP, and VAP-1 levels—is not behind the elevated VAP-1 levels, and therefore, sVAP-1 cannot be considered as a general inflammation marker (3). Moreover, for example, in inflammatory bowel disease, no increase in VAP-1 levels has been found (64). In obesity, variable effects on VAP-1 concentrations have been reported in different cohorts. In the Cardiovascular Risk in Young Finns study consisting of 2183 persons, a negative correlation between the VAP-1 concentration and the body mass index among women was seen. No correlation was obtained in men (3). In another study, no correlation between VAP-1 levels and body mass index was seen among 2206 healthy adolescents (37), However, in morbid obesity, a direct correlation was observed among 74 nondiabetic persons (135).

**Table 3.** Soluble Vascular Adhesion Protein-1 Levels in Different Human Diseases

	*Comments*	*References*
Elevated		
Diabetes	Elevated both in type 1 and 2	
	In type 1 correlates to late complications (*n* = 287, *p* < 0.001, *r* = 0.27)	(16)
	In type 2 predicts end-stage renal disease (*n* = 604, HR 1.55, 95% CI 1.12–2.14, *p* < 0.01)	(70)
	Cardiovascular mortality (*n* = 661, HR 5.83, 95% CI 1.17–28.97)	(69)
	Cancer mortality (*n* = 568, HR 2.95, 95% CI 1.31–6.63, *p* = 0.009)	(148)
Liver diseases^a^	Elevated in several chronic liver diseases	
	Values correlate to severity of the liver injury in NAFLD (*n* = 144 *p* = 0.001 for fibrosis in multivariate analysis)	(136)
	No change in paracetamol poisoning and primary sclerosing cholangitis	(65)
Skin diseases	Psoriasis	
	Correlates to pruritus (*n* = 71, *p* = 0.03)	(80)
Congestive heart failure	Increases with severity (*n* = 271, *p* < 0.0001)	(17)
Multiple sclerosis	Correlates with inflammatory activity measured by MRI (*n* = 66, *p* = 0.0068)	(5)
Alzheimer's disease	Correlates with severity (*n* = 135, *p* < 0.001)	(130)
Hemorrhagic stroke	Predicts outcome (66 patients, *p* = 0.001)	(39)
Cancer	Gastric cancer	
	Low values predict poor outcome (*n* = 107, *p* = 0.011)	(143)
	Colorectal cancer	
	Low values predict poor outcome (*n* = 100, *p* = 0.034)	(129)
	High values predict cancer mortality (*n* = 300, HR 1.003, 95% CI 1.0001–1.0050, *p* < 0.05)	(75)
	Hepatocellular cancer (*n* = 55, *p* < 0.01 compared to cirrhosis)	(56)
Organ transplantation	Heart	
	Correlates with left ventricular diameter and immunosuppressive medication in heart transplantation (*n* = 128, *p* < 0.05)	(61)
	Kidney (*n* = 130, *p* < 0.05 compared to controls)	(61)
Chronic kidney disease	Associates with severity (*n* = 262, OR 1.63, *p* = 0.018)	(77)
Systemic sclerosis	High levels correlate with lower frequency and severity of interstitial lung disease (*n* = 71, *p* < 0.05)	(141)
Decreased		
Cancer	Thyroid, correlates negatively to serum thyroglobulin levels (*n* = 57, *p* < 0.001)	(42)

Only studies involved more than 50 patients have been selected and population-based studies have been explained in the text.

^a^ Certain subgroups of liver diseases smaller than 50.

CI, confidence interval; HR, hazard ratio; MRI, magnetic resonance imaging; NAFLD, nonalcoholic fatty liver disease; OR, odds ratio.

In general, the protein concentration of sVAP-1 corresponds well to the level of SSAO enzymatic activity found in plasma or serum samples. The highest sVAP-1 levels are usually found in patients with metabolically compromised type 1 diabetes and in patients suffering from chronic liver diseases (65). Intravenous glucose tolerance and hyperinsulinemic clamp tests revealed that glucose as such does not increase the sVAP-1 levels. Instead, the levels are inversely correlated with insulin concentrations (115).

In atherosclerotic women, increased level of sVAP-1 correlates directly with intima–media thickness and carotid plaques already in a subclinical disease in models adjusted for the known risk factors (3). In glucose tolerance tests, individuals with increased carotid intima–media thickness had more significant change in their sVAP-1 concentrations than people with normal thickness (71). Moreover, the VAP-1 concentration in plasma predicts major adverse cardiovascular effects (*e.g.*, myocardial infarction and stroke) and cardiovascular mortality in fully adjusted models in a general population. In addition, inclusion of the sVAP-1 level into the Framingham model of cardiovascular risk factors improved the risk discrimination and the reclassifying performance of the model (2, 3). This suggests that sVAP-1 might be a marker of future risk of major adverse cardiovascular effects in apparently healthy persons.

In line with these observations, Boomsma *et al.* (15) have showed that sVAP-1 concentration is an independent prognostic marker for mortality in chronic heart failure among the patients suffering from that disease. Similarly, in patients suffering from type 2 diabetes, the sVAP-1 levels predict 10-year all-cause, cardiovascular and cancer mortalities and independently improve the risk predictions above the well-established risk factors (69). Interestingly, VAP-1 mRNA expression is increased in atherosclerotic aortic valves (6), and therefore, VAP-1 may be involved in leukocyte trafficking to the developing lesions. sVAP-1 may contribute to the atherogenesis also by producing hydrogen peroxide, which at low concentrations has multiple signaling effects, and at high concentrations is directly cytotoxic to endothelial cells. Similarly, the aldehydes produced by the catalytic activity of VAP-1 can be damaging and involved in the generation of advanced glycation end-products (125).

### Imaging

**FIG. 6.**
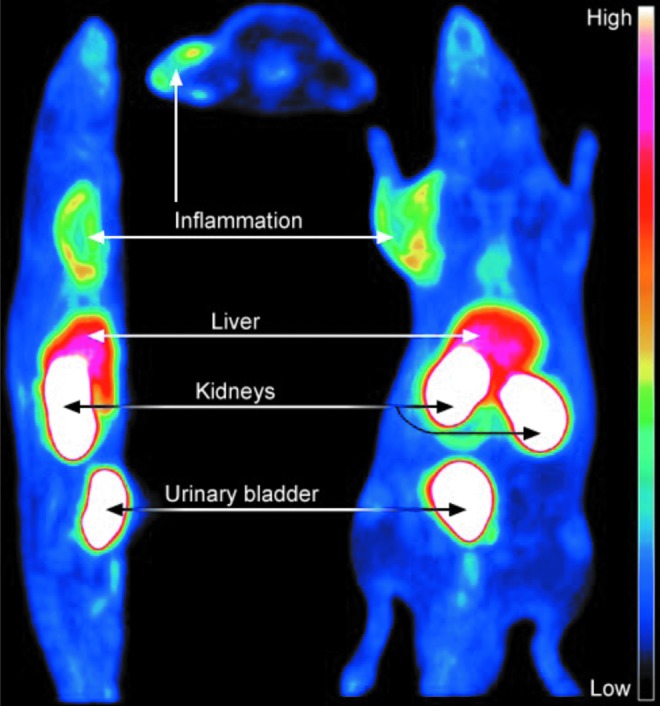
**VAP-1 can be used as a target for imaging.** Representative sagittal (*left*), transaxial (*middle*), and coronal (*right*) multiplane PET images of [^18^F]FDR-Siglec-9 (VAP-1 ligand) biodistribution in a rat. The images are summation from 10 to 60 min postinjection. Reproduced with permission from Chemical Communication [from Li *et al.* (73)].

As VAP-1 is rapidly translocated from the intracellular vesicles onto the vascular surface on inflammation, it is easily accessible by intravascularly administered imaging agents. The proof-of-concept studies using different experimental inflammation models were performed already in late 1990s and early 2000 with monoclonal antibodies against VAP-1 (47). After the crystallographic structure of VAP-1 was resolved and the enzymatic nature of VAP-1 with a narrow substrate channel became evident, peptides fitting to the enzymatic groove were designed and used for imaging. These labeled peptides demonstrated that inflammation can be imaged by targeting VAP-1 (10, 67). However, further development was triggered when the leukocyte ligands of VAP-1, Siglec-10, and Siglec-9 were discovered (1, 59). A peptide from Siglec-9 fitting to the enzymatic groove of VAP-1 has shown its imaging power both as ^18^F and ^68^GA-DOTA conjugates (73, 74, 122) (Fig. 6). Based on these preclinical results, VAP-1 seems to be a potential imaging target for the localization of an inflammatory process also in clinical settings.

### VAP-1 as a potential therapeutic target

Due to the efficacy of VAP-1 inhibitors and anti-VAP-1 antibodies in a wide variety of different inflammatory diseases and cancers in experimental animal models, they have recently become a popular target with high expectations in the pharmaceutical industry.

#### Antibodies

Biotie Therapies (currently Acorda) and its collaborators have been pioneers in developing antibodies against VAP-1 (57). Its fully human anti-VAP-1 antibody (BTT1023, also known by name Timolumab) has been well tolerated and shown beneficial effects both in psoriasis and rheumatoid arthritis. More specifically, the placebo controlled study in rheumatoid arthritis patients (*n* = 24) demonstrated significant improvements in disease activity score among patients receiving repeated BTT1023 infusions (at a dose of 8 mg/kg). Less robust improvements in the clinical score were seen in the psoriasis trial. However, several patients also in this Phase 1b trial seemed to benefit from the treatment. Currently, the antibody is in a Phase IIa clinical trial for primary sclerosing cholangitis, a progressive, liver-destroying, immune-mediated biliary disease characterized by bile duct inflammation and fibrosis. This trial is an investigator-sponsored, open-label, single-arm, multicenter study evaluating the safety, pharmacokinetics, and efficacy of BTT1023 treatment (7).

#### Small-molecule inhibitors

Astellas, Pharmaxis, and Promaxigen have developed small molecular inhibitors against VAP-1. Due to recent acquirements and licensing deals, Pharmaxis VAP-1 project is further developed by Boehringer-Ingelheim and Promaxigen develops its project together with Roche. Astellas VAP-1 inhibitor (ASP8232) is in Phase II trial for diabetic macular edema in the United States and for diabetic nephropathy in Europe. Pharmaxis, developed its inhibitor (PXS4728A) through Phase 1 clinical studies, which demonstrated it to be safe, well tolerated, orally bioavailable, and to cause long-lasting target inhibition. The future primary target in Boehringer-Ingelheim trials will be nonalcoholic steatohepatitis, a progressive form of nonalcoholic fatty liver disease. It is a common consequence of obesity and type 2 diabetes, the frequency of which is dramatically increasing in the Western world and can lead to irreversible liver damage. Possible other targets of Boehringer-Ingelheim will be COPD and other diseases with unmet medical need (132a). Promaxigen's inhibitor PRX167700 is currently in Phase II clinical trials for inflammatory pain (4a).

## Conclusions

The function of VAP-1 in leukocyte trafficking has been convincingly shown *in vitro* and *in vivo* using both pharmacological and genetic tools. There is also strong evidence to support the concept that many of the VAP-1 effects are dependent on its enzymatic activity. However, the interplay between the enzyme activity-dependent and enzyme activity-independent functions of VAP-1 in leukocyte trafficking needs to be dissected even in more detail at the mechanistic level in the future. In particular, the identity of different VAP-1 substrates and ligands on the leukocytes, the role of putative aldehyde products on leukocytes during the adhesion cascade, and the signaling effects of the generated hydrogen peroxide both on the leukocytes and endothelium should be addressed. Similarly, there is an urgent need to understand which effects of VAP-1 in fibrosis, angiogenesis, and cancer are secondary to altered leukocyte trafficking, and which are direct effects on endothelial cells, smooth muscle cells, pericytes, and myofibroblasts. Similarly, the physiological VAP-1 substrates in different tissues and the physiological role of VAP-1 in the metabolic control are far from clear at the moment.

Translation of VAP-1 inhibition for clinical purposes also heavily depends on more detailed understanding of the VAP-1 functions. In addition, the value of VAP-1 targeting in different disease models should increasingly be analyzed in clinically relevant therapeutic, rather than prophylactic, settings. Moreover, since several small-molecule VAP-1 inhibitors appear not to be completely specific for VAP-1, it would be useful to confirm the VAP-1 dependency of the beneficial therapeutic responses using VAP-1-deficient and VAP-1 enzyme activity-deficient mice. In addition, the potency of VAP-1 inhibition by antibodies and SSAO inhibitors should be directly compared in relevant disease models. Finally, the potential value of sVAP-1 as a promising disease biomarker in selected pathologies would warrant testing of the usability of sVAP-1 measurements in actual patient stratification or even as a diagnostic and prognostic tool in individual patients.

In conclusion, VAP-1 plays an important role in a multitude of inflammatory conditions by regulating leukocyte infiltration into the affected tissues. Increased VAP-1 expression and luminal VAP-1 translocation take place in many acute and chronic inflammations both in animal models and in patients. Neutralizing anti-VAP-1 antibodies and small-molecule VAP-1 inhibitors have been successfully utilized in experimental models to reduce adverse inflammatory reactions, among others, in acute skin, lung, liver, and eye inflammation, in chronic joint, gut, and brain inflammation, in severe systemic infections, in ischemia/reperfusion injury in different organs, in liver, lung, and kidney fibrosis, and in cancer. The reduction of inflammation and tissue pathology in several different settings by VAP-1 blockade is consistent with the observations that many different leukocyte subtypes, including neutrophils, monocytes, and lymphocytes, can utilize VAP-1 in the extravasation process. Eventually, data from the ongoing clinical trials will show whether the potential of VAP-1 targeting in inhibiting inappropriate inflammation can be exploited in the clinics.

## Abbreviations Used

AOC: amine oxidase copper containing
ConA: concanavalin A
COPD: chronic obstructive pulmonary disease
HEV: high endothelial venule
HUVECs: human umbilical vein endothelial cells
IL: interleukin
LOX: lysyl oxidase
LPS: lipopolysaccharide
mAb: monoclonal antibody
MDSC: myeloid-derived suppressor cells
mRNA: messenger RNA
NOD: nonobese diabetic
Siglec: sialic acid-binding immunoglobulin-type lectins
siRNA: small interfering RNA
SSAO: semicarbazide-sensitive amine oxidase
sVAP-1: soluble VAP-1
TNF: tumor necrosis factor
VAP-1: vascular adhesion protein-1
